# Photosynthetic Metabolism and Nitrogen Reshuffling Are Regulated by Reversible Cysteine Thiol Oxidation Following Nitrogen Deprivation in Chlamydomonas

**DOI:** 10.3390/plants9060784

**Published:** 2020-06-23

**Authors:** Amanda L. Smythers, Evan W. McConnell, Hailey C. Lewis, Saher N. Mubarek, Leslie M. Hicks

**Affiliations:** Department of Chemistry, The University of North Carolina at Chapel Hill, Chapel Hill, NC, 27599, USA; asmyther@email.unc.edu (A.L.S.); evanmcc@live.unc.edu (E.W.M.); hlewis13@live.unc.edu (H.C.L.); saher@live.unc.edu (S.N.M.)

**Keywords:** *Chlamydomonas*, nitrogen deprivation, proteomics, oxidative signaling, stress, photosynthesis

## Abstract

As global temperatures climb to historic highs, the far-reaching effects of climate change have impacted agricultural nutrient availability. This has extended to low latitude oceans, where a deficit in both nitrogen and phosphorus stores has led to dramatic decreases in carbon sequestration in oceanic phytoplankton. Although *Chlamydomonas reinhardtii*, a freshwater model green alga, has shown drastic systems-level alterations following nitrogen deprivation, the mechanisms through which these alterations are triggered and regulated are not fully understood. This study examined the role of reversible oxidative signaling in the nitrogen stress response of *C. reinhardtii*. Using oxidized cysteine resin-assisted capture enrichment coupled with label-free quantitative proteomics, 7889 unique oxidized cysteine thiol identifiers were quantified, with 231 significantly changing peptides from 184 proteins following 2 h of nitrogen deprivation. These results demonstrate that the cellular response to nitrogen assimilation, photosynthesis, pigment biosynthesis, and lipid metabolism are regulated by reversible oxidation. An enhanced role of non-damaging oxidative pathways is observed throughout the photosynthetic apparatus that provides a framework for further analysis in phototrophs.

## 1. Introduction

There is evidence to support that anthropogenic climate change will have serious effects on global temperatures, extreme weather events, and nutritional availability, demanding rapid and far-reaching action to reduce greenhouse gas emissions while also improving global carbon sequestration [1,2]. Although oceanic phytoplankton are responsible for approximately half of the global carbon fixation, nutrient-depleted conditions limit their biomass production and subsequent carbon sequestration [3,4]. Surface waters between 40° S and 40° N, commonly known as the low latitude oceans, are becoming too nutrient deplete to foster phytoplankton; these waters are now responsible for over two thirds of CO_2_ outgassing to the atmosphere, thus becoming one of the major contributors to climate change [5,6]. Furthermore, these oligotrophic “nutrient-deserts” are growing. From 1998 to 2006, they increased in size by 6.6 × 10^6^ km^2^, or 15% [7,8]. The effects of future decreases in nutrient availability will not only be limited to phytoplankton; agricultural crops, most of which already rely on synthesized nitrogen sources, store approximately 75% of their leaf-based nitrogen stores in their chloroplast, demonstrating the need for serious examination of photosynthetic pathways for deprivation acclimation [9].

*Chlamydomonas reinhardtii* is a unicellular green alga that has been used as a model phototroph for investigating the change in photosynthetic capacity following light fluctuations, heat stress, nutrient deprivation, and other conditions related to climate change [10,11,12,13,14,15,16]. Nitrogen deprivation in particular has been studied extensively in *C. reinhardtii* due to its ability to increase neutral lipid production, with a 200% triacylglycerol (TAG) increase generated in the 24 h following nitrogen deprivation [17,18,19]. These TAGs can be transesterified to produce a drop-in ready biodiesel; optimization of this biosystem may provide long-term fossil fuel replacement [20]. However, nutrient deprivation produces undesirable phenotypes, including decreases in photosynthetic activity as well as arrest of protein translation and cell division [17]. Thus, the significance of nitrogen deprivation is twofold: the need to understand phototrophic acclimation to nutrient deficiency combined with the goal to increase the feasibility of algal-based biofuels makes this a key strategy in addressing and alleviating global climate change.

The metabolic activity of *C. reinhardtii* is highly regulated by a portfolio of post-translational modifications (PTMs), including phosphorylation, glycosylation, and acylation [21,22,23,24,25]. Reversible oxidation is among the least understood PTM in phototrophic eukaryotes; although several studies have mapped the reversibly oxidized proteome of *Chlamydomonas*, there are still gaps differentiating the use of reversible oxidation for signaling and activity modulation and irreversible oxidation as markers for permanently damaged proteins [26,27,28,29,30,31]. While irreversible oxidation is associated with large scale protein damage, reversible oxidation, particularly of cysteine thiols, acts as a protein switch similar to that of phosphorylation, in which temporary oxidation may modulate protein activity [32]. Reversible oxidation in the form of disulfide bonds, *S*-glutathionylation, *S*-nitrosylation, and *S*-sulfonation has been implicated in the regulation of signaling, stress response, transcription, translation, and carbon metabolism, as well as photosynthesis [33,34,35,36,37]. While nitrosylation, glutathionylation, and the thioredoxome have been explored in *C. reinhardtii*, the extent to which oxidative signaling is used to respond and acclimate to nitrogen deprivation is poorly understood [26,27,28].

**Figure 1:**
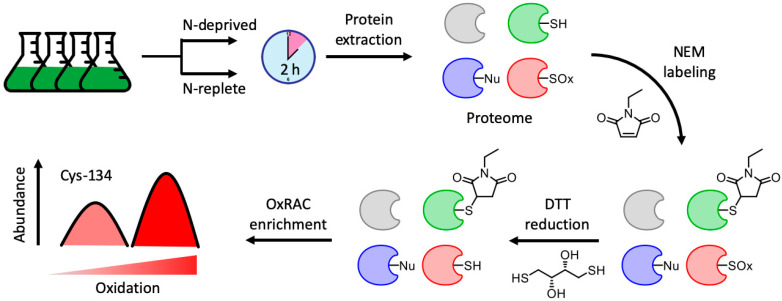
General workflow for nitrogen deprivation paired with redox enrichment. Two groups of four biological replicates of *Chlamydomonas reinhardtii* were grown to mid-exponential phase before exchanging the media to be either nitrogen replete or deplete. Cells were harvested after 2 h and subjected to the oxidized cysteine resin-assisted capture (OxRAC) protocol, through which reduced cysteine thiols are labeled with *N*-ethylmaleimide (NEM), a thiol-specific (-SH) alkylating agent with limited reactivity toward oxidized cysteines (-SOx) and other nucleophilic residues (-Nu). Reversibly oxidized residues are then enriched via a Thiopropyl Sepharose (TPS6B) resin before label free quantification on a mass spectrometer.

In this study, the oxidative response in *C. reinhardtii* was evaluated following nitrogen deprivation using an enrichment-based, label-free quantitative proteomics approach (Figure 1). Oxidized cysteine thiols were quantified following 2 h of nitrogen deprivation, revealing significant changes in chlorophyll biosynthesis, amino acid metabolism, and transcription and translation pathways. This analysis demonstrates that the oxidative environment of the cell is impacted by nitrogen deprivation and used to regulate the resulting metabolic processes.

## 2. Results and Discussion

### 2.1. Global and Redox-Enriched Proteomic Coverage

While reversible oxidative changes in *C. reinhardtii* and the model plant species *Arabidopsis thaliana* have been identified after as little as 10 min, physiological changes under nitrogen deprivation take much longer [29,38]. Preliminary experiments showed robust redox changes following 2 h of nitrogen deprivation (data not shown), thus this timepoint was chosen to examine the role of reversible oxidation in downstream processes that occur in phototrophs. A complementary global differential proteomic analysis was performed to normalize changes in oxidation with changing protein abundances. However, none of the 2853 proteins quantified in the global study revealed a 2-fold protein abundance change after 2 h of treatment (Supplemental Table S1). While a previous study reported changes in 106 proteins under identical treatment conditions, this was in a cell-wall deficient strain (CC-400 cw15 mt^+^) and used a lower significance threshold of 1.2 [17]. The higher significance threshold and the additional protection of the cell wall, a critical defense barrier against stressors, may account for the observed differences in this experiment.

The oxidized cysteine resin-assisted capture (OxRAC) method uses rapid and efficient *N*-ethylmaleimide (NEM) alkylation paired with thiol-disulfide exchange chromatography to enrich for exposed cysteine-containing residues via a Thiopropyl Sepharose (TPS6B) resin and has been used to show cell-encompassing changes in protein oxidation following stress exposure [39]. Using this method, 9295 oxidized cysteine sites were quantified on 7889 unique peptides from 3395 proteins across nitrogen deprived and replete samples (Supplemental Table S2). A negative control, wherein samples were not reduced prior to resin enrichment, revealed only 170 unique proteins, thereby suggesting efficient NEM alkylation. Furthermore, none of the proteins identified in the negative control contained oxidized cysteine sites that met the significance threshold in the redox-enriched dataset. Quantified peptides included up to six oxidized cysteine residues, though the overwhelming majority featured only one site (91%). This is expected; while 93% of proteins in the *C. reinhardtii* proteome contain cysteine residues, cysteines constitute only 1.6% of protein sequences. Therefore, tryptic peptides containing more than one cysteine are uncommon, with an *in silico* analysis of the *C. reinhardtii* protein database identifying 79% and 16% of potential cysteine-containing tryptic peptides having one and two Cys residues, respectively.

**Figure 2:**
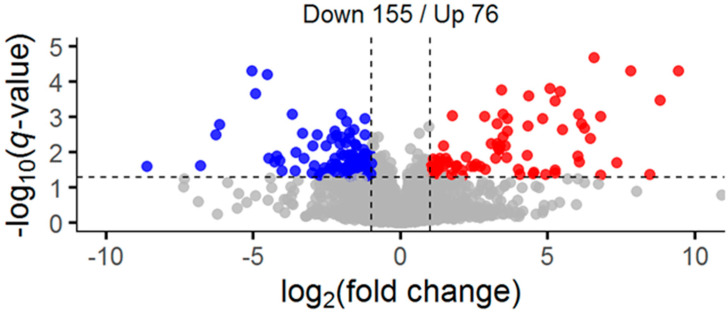
Analysis of significant oxidative changes in nitrogen deprived and nitrogen replete *C. reinhardtii* samples. Volcano plot of two-tailed equal variance *t*-tests between conditions following a Benjamini–Hochberg false discovery rate adjustment. Blue denotes significantly decreased identifiers and red denotes significantly increased identifiers.

These results revealed 231 unique peptides from 185 proteins to contain significantly different reversible oxidation between the nitrogen-deprived and replete condition, with 155 decreased and 76 increased (Figure 2). Fold changes were converted to log_2_FC (FC_l2_) for ease of comparison and are reported as such hereafter. Cysteines found to decrease in oxidation may be the result of either thiol reduction or increased oxidation to an undetectable irreversible oxidized form. Despite this ambiguity, the utility of probing the entire cysteine thiol redoxome to reveal reactive cysteines and generate hypotheses for validation is irrefutable. Cross-comparison of the 185 proteins identified here with those found with specific oxidative modifications in prior studies include 4 nitrosylated, 3 glutathionylated, 6 both nitrosylated and glutathionylated, and 20 proteins targeted by thioredoxin (Supplemental Table S3) [27,28,37,40]. Thus 88% of the 185 unique proteins with significantly different oxidation sites identified in this study have not been characterized previously.

**Figure 3:**
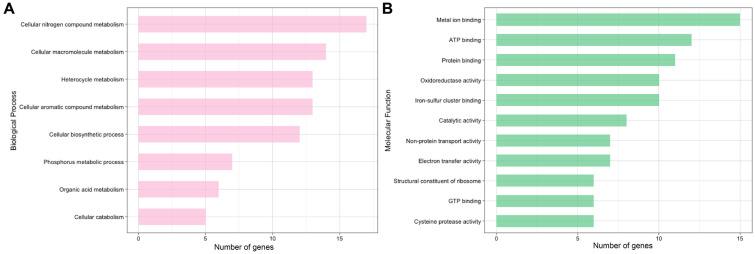
*C. reinhardtii* was subjected to nitrogen deplete or replete conditions before harvesting and quantifying changes in reversible oxidation via LC–MS/MS. Proteins with significantly different oxidation sites were annotated using UniProt and the Panther database. All gene ontology terms with at least five genes describing (**A**) biological processes and (**B**) molecular function are plotted in descending order.

Of the significantly different oxidized cysteines quantified, 94% of the proteins were also measured in the global samples, demonstrating that significant changes in oxidation were not a result of overall abundance changes due to protein synthesis/turnover. Annotation revealed differentially oxidized cysteines in pathways relating to chlorophyll biosynthesis, amino acid metabolism, chloroplastic regulation and photosynthesis, and protein transcription and translation, supporting a significant role of oxidative signaling in the nitrogen deprivation response (Figure 3). 

### 2.2. Nitrogen Assimilation and Allocation

**Figure 4:**
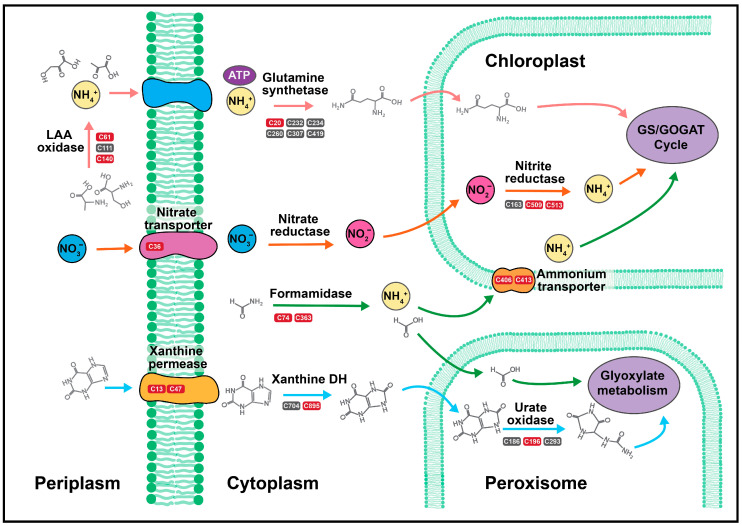
*C. reinhardtii* was subjected to nitrogen deplete or replete conditions before harvesting and quantifying changes in reversible oxidation via LC–MS/MS. Major nitrogen allocation pathways regulated via oxidative signaling are presented. Red cysteine residues indicate increased oxidation, while blue cysteine residues indicate decreased oxidation. Grey cysteine residues were found in the dataset but did not significantly change under nitrogen deprivation.

Ten proteins relating to nitrogen assimilation/allocation were found to have significantly different cysteine oxidation, with a total of 14 cysteine sites across 13 peptides increased in oxidation (Table 1, Figure 4). The greatest increase in oxidation was on xanthine/uracil permease (Cre10.g442800.t1.1), which had increases of FC_l2_ 5.5 and 8.5 on C13 and C47, respectively.

Xanthine/uracil permease is a component of the purine degradation pathway encoded by *XUV6*, which increases in expression during the TAG synthesis phase following 6–24 h of nitrogen deprivation [41]. However, within the first 2 h of deprivation, RNA catabolism begins to increase nitrogen stores for protein synthesis [17]. Thus the oxidation of xanthine/uracil permease may increase activity prior to genetic upregulation, as cells seek to increase the flux of nitrogen accumulation during early starvation. As this oxidation has never been characterized, further validation is needed to determine its effect on overall activity.

The second largest increase in oxidation was a 7.8 FC_l2_ on C74 of formamidase (Cre16.g691750.t1.2), a hydrolase that catalyzes the reaction of formamide and water to produce formate and ammonia (Figure 4). Formamidase also had a 3.4 FC_l2_ increase in oxidation on C363. Previous work has shown that *C. reinhardtii* prefers ammonium as its inorganic source of nitrogen and that it releases ammonia from other nitrogen stores when under nutrient stress within one hour of nitrogen deprivation [16]. While the oxidation of formamidase has not been shown previously in any organism, the extensive increase in oxidation on C74 suggests a potential role of cysteine regulation for enhanced activity; however further validation is needed to determine this role.

Similarly, two cysteine residues on L-amino acid oxidase isozymes, C61 (Cre12.g551352.t1.1) and C140 (Cre12.g551353.t1.1), demonstrated significant increases in oxidation, with FC_l2_ of 6.1 and 6.4, respectively (Table 1). Interestingly, neither of these cysteine residues are conserved across the two isozymes, making each oxidation site unique to each enzyme. L-amino acid oxidase catalyzes the periplasmic deamination of free amino acids, releasing an α-keto acid, ammonia, and hydrogen peroxide, and has been shown to act as a nitrogen scavenger during nitrogen deprivation to enable rapid assimilation of nitrogen following extracellular amino acid availability [41,42,43]. Although the coding gene in *C. reinhardtii*, *LAO1*, has been previously shown to have the largest increase of transcript-level nitrogen-assimilation genes 48 h following nitrogen deprivation [16,42,43], the protein abundance, measured by the quantification of 35 individual peptides (3 unique), was not upregulated in this study (*p* = 0.22; Supplemental Table S1). More important, this is the first evidence linking reversible oxidation to its regulation.

The plastid ammonium transporter (Cre13.g569850.t1.2), encoded by *AMT4,* had a 6.8 FC_l2_ on C406 and a 4.3 FC_l2_ on C413, indicating a potential role for oxidative regulation on ammonium movement traversing the chloroplast (Figure 4). Ammonium transporters in *C. reinhardtii* are post-translationally modified by nitric oxide (NO), in which increased oxidation results in inhibition of ammonia transport [44]. Both the expression of *AMT4* and the abundance of NO signaling increases within 2 h following nitrogen deprivation, supporting a link between NO oxidation of the plastid ammonia transporter and the hallmark degradative pathways initiated in the chloroplast following nitrogen starvation [16,17,45]. Although this study did not see global abundance increases (quantified by three unique peptides) and cannot discriminate the presence of specific oxidative modifications, the high fold changes in oxidation suggest a similar phenomenon. Furthermore, the oxidation of the plastid ammonia transporter is likely due to a downregulation of nitrogen flux into the chloroplast; chloroplastic protein translation and chlorophyll synthesis decreases following nitrogen deprivation, thus plastid-localized nitrogen stores would not be needed [46]. Rather than immediately dismantle the ammonium transporter, the oxidation may serve as a thiol switch that can modulate activity based on nitrogen availability. Nitrogen deprivation also produced a 6.0 FC_l2_ on C36 on the nitrate transporter (Cre09.g410850.t1.2) and a 4.3 FC_l2_ on C509 and C513 of nitrite reductase (Cre09.g410750.t1.2), both of which have been implicated in NO-induced functional inhibition (Table 1) [44].

Inorganic stores of nitrogen are assimilated into proteins via the regulatory glutamine synthetase [16]. While this enzyme has been shown to also be modified by NO in *Medicago truncatula* nodules, resulting in tyrosine nitration, in vivo and *in vitro* studies indicate that NO does not change the activity of glutamine synthetase in *C. reinhardtii* [44,47]. However, in this study, glutamine synthetase (Cre02.g113200.t1.1) displayed increased oxidation on C20 (FC_l2_: 5.2) following 2 h of nitrogen deprivation (Figure 4). This oxidation, combined with a decrease in free amino acids and overall arrest of protein synthesis, suggests that while not impacted by nitrosylation, the activity of glutamine synthetase may be decreased through oxidative mechanisms [17]. Both the C196 of urate oxidase (Cre12.g504950.t1.2) and C895 of xanthine dehydrogenase (Cre12.g545101.t1.1) also increased in oxidation, with FC_l2_ of 1.3 and 4.3, respectively; urate oxidase has also been shown to contain glycosylation and nitrosylation sites [27,37]. While it is possible that the flux of nitrogen allocation into amino acid production may be regulated via increases in oxidation, further studies are necessary to determine the impact of individual cysteine sites.

### 2.3. Photosynthesis and Chloroplastic Regulation

The classic model of photosynthetic oxidation counters reactive oxygen species (ROS) production against antioxidants in a balancing act, in which a shift towards increasing ROS is indicative of either cellular damage, decreasing antioxidant capacity, or both [30,48]. However, recent studies, including those quantifying NO- and glutathione-specific oxidation, suggest ROS formation and subsequent signaling is a complex regulatory process in which oxidative signals are transmitted both within and outside of the chloroplast [26,45,49,50]. Although nitrogen deprivation leads to a decrease in photosynthetic flux paired with the degradation of the photosynthetic apparatus and the ultrastructure of the chloroplast, this damage does not occur until >10 h following deprivation [17,46]. Thus, the 25 significantly differenet identifiers in this study related to photosynthetic and chloroplastic function may be related to signaling rather than damage (Supplemental Table S4).

Previous research has shown minimal decreases to photosynthetic efficiency following 2 h of nitrogen deprivation; therefore, it was not surprising that only 12 Cys-sites were found directly effecting electron transfer [16,17,46] (Table 2). Changes were observed in the light-harvesting protein of photosystem I, five paralogs of ferredoxin, zeaxanthin epoxidase, and chloroplastic thioredoxin. Both the light harvesting complex of photosystem I (*LHCA8*) and zeaxanthin epoxidase (*ZEP1*) decreased in oxidation by a FC_l2_ of −1.3, occurring on C24 and C520 respectively. Zeaxanthin epoxidase catalyzes the conversion of zeaxanthin, a radical scavenger, to violaxanthin, increasing photon flux through the light harvesting antenna complexes and decreasing non-photochemical quenching (NPQ) [51]. Decreases in both photosystem I-associated fluorescence and NPQ have been shown within the first six hours of nitrogen deprivation despite an overall increase in zeaxanthin, suggesting that the decrease in NPQ is likely due to an overall down-regulation in photosynthesis rather than an increased photon flux toward photosynthetic reaction centers [46,52]. Therefore, although these modifications have not been characterized, it is possible that they are involved in the initial signaling for shifting light capture prior to photosystem degradation, with cysteine reduction occurring in a reducing environment following nitrogen deprivation.

**Figure 5:**
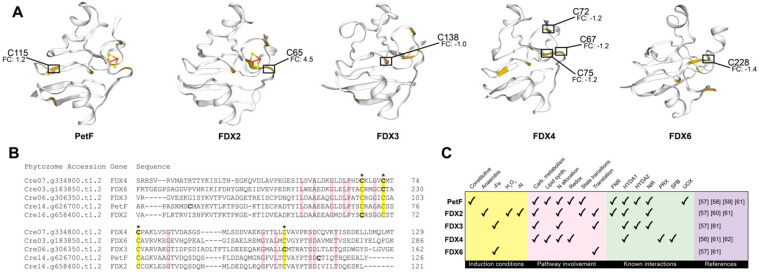
Oxidative changes across the ferredoxin network, observed following 2 h of nitrogen deprivation in *C. reinhardtii*. (**A**) The structures of the five ferredoxin isoforms quantified in this study, modeled using ExPASy. Cysteine residues are shown in yellow. PetF is missing one significantly changing cysteine site (C30, FC_l2_: −1.6) due to limitations in the modeling algorithms. All fold changes (FC) indicate the log_2_FC. (**B**) Sequence alignment of the five ferredoxin isoforms, performed using Clustal Omega. Yellow shading denotes conserved cysteines while pink shading denotes other conserved residues. Residues required for 2Fe-2S coordination are marked with an asterisk. Cysteines significantly decreased in oxidation are shown in bold while those significantly increased in oxidation are shown in bold and underlined. (**C**) A summary of the known function(s) of the five ferredoxin isoforms quantified in this study. Abbreviations: FNR, ferredoxin—NADP(+) reductase; HYDA1/HYDA2, Fe-Fe hydrogenase; NiR, nitrite reductase; PRX, peroxiredoxin; SPB, squamosa promoter-binding protein; and UOX, urate oxidase.

The primary photosynthetic ferredoxin, encoded by *PetF*, transports electrons from photosystem I to ferredoxin-NADP+ reductase, generating NADPH that can be used to fuel the Calvin–Benson–Bassham cycle [53]. Following 2 h of nitrogen deprivation, ferredoxin had two differentially oxidized cysteines, C30 and C115, with FC_l2_ of −1.6 and 1.2, respectively (Figure 5). Additionally, four other ferredoxins, including those encoded by *FDX2, FDX3*, *FDX4*, and *FDX6*, had differentially changed oxidized cysteines; FDX4 produced an identifier with three localized cysteine residues (C67, C72, and C75) and a −1.2 FC_l2_ decrease in oxidation, FDX3 had a −1.0 FC_l2_ decrease in oxidation on C138, FDX6 had a −1.4 FC_l2_ decrease in oxidation on C228, and FDX2 had 4.5 FC_l2_ increased oxidation on C65. Structures and sequences of the five isoforms were compared to visualize relationships between cysteines with significant changes in oxidation (Figure 5) [54,55]. Except PetF, all isoforms contained changes in oxidation on at least one of the four cysteines responsible for coordinating the 2Fe-2S catalytic clusters, with FDX4 showing a significant decrease in oxidation on three of the coordinating cysteines [56]. FDX2 was the only isoform that showed an increase in oxidation on one of the 2Fe-2S coordinating cysteines.

Although PetF is the most prevalent ferredoxin with >95% of the expressed protein, all of the ferredoxins contribute to environmental acclimation in *C. reinhardtii* (Figure 5C) [57]. Under nitrogen deprivation, this includes contributing to [Fe-Fe]-hydrogenase (*HYDA1*) mediated hydrogen production; both PetF and FDX2 contribute to H_2_ production by shuttling electrons between pyruvate:ferredoxin oxidoreductase and [Fe-Fe]-hydrogenase [58,59]. Following this study’s 2 h of nitrogen deprivation, [Fe-Fe]-hydrogenase had three cysteines, C88, C225, and C238, decreased in oxidation by FC_l2_ of −1.6, −2.2, and −2.6, respectively. However, hydrogenases are oxygen-sensitive; since *C. reinhardtii* does not experience a significant loss in oxygen production until at least 5 h of nitrogen deprivation, it is unlikely that the decrease in oxidation is directly related to fermentative hydrogen production [46,58,60]. Additionally, while PetF, FDX2, and FDX3 have been implicated in nitrogen allocation due to their abilities to donate electrons to nitrite reductase, nitrite reductase preferentially interacts with FDX2, demonstrating that *FDX2* encoded ferredoxin, whose transcripts are upregulated following nitrogen deprivation, plays an enhanced role in nitrogen assimilation and allocation following deprivation [57,61]. Over-oxidation is predicted to damage FDX2 beyond function. While transcripts of FDX2 increase in cells following exposure to 1 mM hydrogen peroxide, protein abundance decreases, with nearly total loss by 2 h post-exposure [57]. Further in vivo experiments are needed to determine the extent to which intracellular oxidation enhances or inhibits overall FDX2 function.

In addition to PetF, previous studies have suggested FDX4 involvement in redox regulation; FDX4, a thioredoxin target, interacts with both peroxiredoxin family protein and squamosa promoter binding protein (SPB), which affects state transitions and photosystem recovery pathways on the post-translational and transcriptional level, respectively [25,28,61,62]. Squamosa promoter binding protein, which has been previously shown to increase in transcripts within the first 30 min of nitrogen deprivation, is also partly responsible for the upregulation of TAG accumulation, with *Chlamydomonas* knockouts accumulating 50% less triacylglycerols during nitrogen-deprivation than those with active SPB [63]. Interestingly, our results show a 1.89 FC_l2_ increase on C1829 of SPB (Cre16.g673250.t1.1; Table 2). This relationship suggests that FDX4 is a more significant player than PetF or FDX2 in the acclimation of *C. reinhardtii* to nitrogen deprivation and should be considered in targeted approaches to increase overall TAG accumulation.

### 2.4. Chlorophyll Biosynthesis

**Figure 6:**
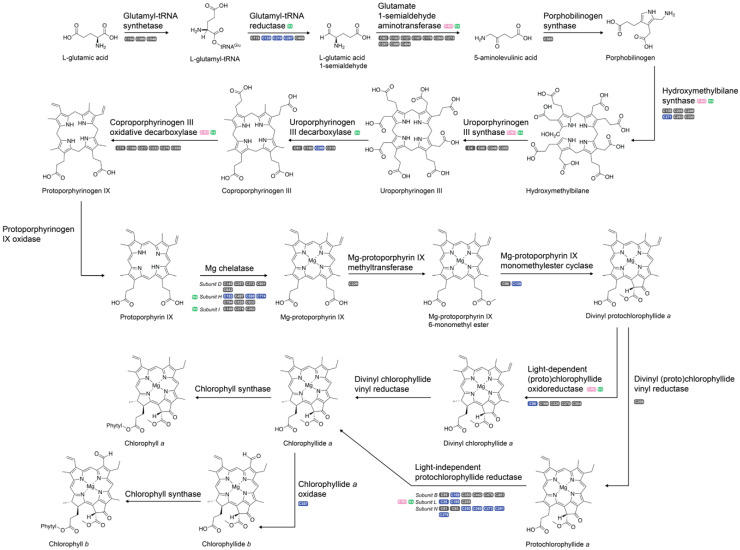
*C. reinhardtii* was subjected to nitrogen deplete or replete conditions before harvesting and quantifying changes in reversible oxidation via LC–MS/MS. The summary of oxidized cysteine sites quantified across the chlorophyll biosynthetic pathway is presented. Blue cysteine residues indicate decreased oxidation, while grey residues were found in the dataset but did not significantly change under nitrogen deprivation. Proteins previously shown to be *S*-nitrosylated are denoted with pink, while those previously shown to be targets of thioredoxin are denoted with green. None of the chlorophyll biosynthesis proteins have been shown to contain glutathionylation.

A decrease in chlorophyll following nitrogen deprivation in *C. reinhardtii* is well established [17,46,64,65,66]. While chlorophyll catabolism has been shown in other algae to recycle nitrogen into high-energy compounds, chlorophyllases and other catabolic enzymes are not upregulated until 24+ h following nitrogen deprivation. Downregulation of biosynthesis-related enzymes, however, occurs within the first few hours [46]. Thus, the decrease in chlorophyll accumulation is assumed to be due to the stagnation of chlorophyll synthesis paired with continuing cellular division and growth, resulting in lower chlorophyll per cell without catabolizing the pigments. However, the dependence on oxidative signaling for downregulation of biosynthetic pathways has thus far remained unexplored. Previous work has shown six proteins of the 17 along the chlorophyll biosynthesis pathway to contain *S-*nitrosylation [27]. Additionally, nine proteins, including all known proteins modified by nitrosylation, are targets of thioredoxin [28]. Following 2 h of nitrogen deprivation, 79 cysteine thiol oxidation sites were quantified across 15 proteins along the chlorophyll synthesis pathway and 19 significantly different oxidation sites on 24 unique peptides were observed across 15 proteins throughout all pigment biosynthesis pathways (Figure 6; Table 3). All but one of the cysteine sites were decreased in oxidation. This data suggests a major role of oxidative regulation on chlorophyll biosynthesis, as glutamyl-tRNA reductase and magnesium chelatase, representing the rate-limiting and chlorophyll committing steps of tetrapyrrole biosynthesis, respectively, each had several significant oxidative changes.

Tetrapyrrole biosynthesis, a precursor to chlorophyll synthesis as well as several non-chlorophyll products (e.g., hemes), begins with activation of glutamate via ligation to tRNA^glu^ by Glu-tRNA synthetase, before the reduction of the α-carboxyl group by glutamyl-tRNA reductase to generate Glu 1-semialdehyde (Figure 6) [67,68,69]. Following 2 h of nitrogen deprivation, three cysteine residues of glutamyl-tRNA reductase (Cre07.g342150.t1.2), C210, C129, and C267, significantly decreased in oxidation, with FC_l2_ of −1.82, −1.67, and −1.19, respectively. Previous experiments showed a decrease in the mRNA of *HEMA*, the gene encoding glutamyl-tRNA reductase, by 2 h post- nitrogen deprivation, preceding changes in transcripts of the photosynthetic apparatus by several hours [16]. However, this was not accompanied by significant changes in protein abundance, as shown both in previous studies as well as the global protein dataset reported herein [16,17]. Both glutamyl-tRNA reductase and glutamate 1-semialdehyde aminotransferase, the latter of which catalyzes the transamination of Glu 1-semialdehyde to form 5-aminolevulinic acid and has been shown to complex with glutamyl-tRNA reductase for efficient substrate channeling, are candidates for thiol-based PTMs, with evidence suggesting modulation via NADPH-dependent thioredoxin reductase C [70,71,72]. Glutamyl-tRNA reductase is also finely regulated in *A. thaliana* via the GluTR-binding protein, FLU protein, a Clp protease, and cpSRP43 in order to precisely control the rate of tetrapyrrole synthesis [73,74,75,76]. However, the mechanism(s) of these interactions have yet to be discerned, despite hypotheses regarding thiol oxidation. This study provides the first experimental evidence of a thiol-based redox switch controlling the initiation of chlorophyll biosynthesis. However, the GluTR-binding protein, FLU protein, Clp protease, or cpSRP43 were found differentially oxidized in redox-enriched samples; ergo, either the enzymatic control of *A. thaliana* and *C. reinhardtii* are vastly different (an unlikely conclusion), or the enzyme(s) responsible for modulating redox occupancy on glutamyl-tRNA reductase is currently unknown. A combination of ROS-targeted enrichment strategies and chemical crosslinking of protein complexes in *C. reinhardtii* under nitrogen deprivation may unveil protein partners contributing to reversible oxidation.

Following the generation of protoporphyrin IX, tetrapyrrole synthesis can either proceed toward chlorophyll synthesis via magnesium chelatase or non-chlorophyll hemes via protoporphyrin IX ferrochelatase, making magnesium chelatase the chlorophyll committing step [73]. Magnesium chelatase is known to be regulated by ATP, Mg^2+^, GUN4 protein, and thioredoxin. Subunit H is one of three subunits of magnesium chelatase and the only subunit with catalytic activity [77]. This subunit contained three cysteines with significantly decreased oxidation, C183 (FC_l2_: −1.8), C598 (FC_l2_: −1.7), and C774 (FC_l2_: −1.8; Figure 6). Despite physiological evidence to support decreased chlorophyll biosynthesis following nitrogen deprivation, studies have shown that reducing cysteines on subunit H of magnesium chelatase increases catalytic activity, suggesting that magnesium chelatase activity is enhanced under nitrogen deprived conditions [78,79]. Chlorophyll biosynthesis requires glutamate availability, thus the substrate load under nitrogen deprivation may be inherently lower. Therefore, although overall biosynthesis is decreased, the rate of activity may be increased as a survival mechanism to maintain cellular homeostasis; the lack of substrate availability would result in lesser accumulation despite increased rates of catalysis. A more targeted flux analysis of glutamate may delineate the balance between activity and accumulation under nutrient deprivation conditions.

Both light-dependent (Cre01.g015350.t1.1) and light-independent protochlorophyllide (NP_958412.2) reductase had considerable changes in oxidation, the former having a −4.5 FC_l2_ on C56 and the latter having eight significantly decreased peptides containing nine cysteines across all three subunits (Table 3). Light-dependent protochlorophyllide reductase is highly sensitive to oxygen levels as well as high light conditions, active only when in the presence of <80 µmol photons m^−2^s^−1^ [80]. Light-dependent protochlorophyllide reductase requires a NADPH electron donor paired with photons to catalyze the reduction of the double bond in the D ring of protochlorophyllide, whereas the light-independent counterpart requires ATP hydrolysis powered by either dithionite or ferredoxin as an electron donor [81,82]. Therefore, a change in the oxidative environment of the chloroplast would likely decrease the activity of the light-independent enzyme, as electron donor availability would be affected. Furthermore, the decrease in oxidation across cysteine residues may be related to the decrease in oxidation on ferredoxin (Section 2.3). Further targeted experiments are needed to discriminate if this is due to reduction or irreversible oxidation.

### 2.5. Lipid Metabolism

Nitrogen deprivation is often studied to examine how algae switch between proliferation and quiescence, resulting in the upregulation and long-term storage of TAGs desired for biofuel production [83]. Although *C. reinhardtii* is not a candidate for large-scale biofuel production due to its low biomass yield and sensitivity to external stressors, it has served as a genetic and molecular model for better understanding photosynthetic lipid production in both algae and plants [84]. In this study, we observed 10 significantly different peptides containing 12 cysteine thiols on eight proteins related to lipid synthesis (Table 4). Among the proteins with significant changes are acetyl CoA synthetase, two fatty acid desaturases, and a phospholipase-B like protein, suggesting that the metabolic restructuring toward TAG synthesis on the post-translational level begins rapidly, despite the fact that the physiological changes do not occur for at least 12–24 h [17].

Following 2 h of nitrogen deprivation, chloroplastic acetyl CoA synthetase (Cre01.g055408.t1.1) had significantly decreased oxidation on C164 and C594, with FC_l2_ of −1.0 and −1.1, respectively. Acetyl-CoA synthetase catalyzes the conversion of acetate into acetyl-CoA and is a known target of thioredoxin as well as prone to nitrosylation [27,40]. However, this study is the first site-specific determination of differential oxidation on acetyl-CoA synthetase and the functional significance of these cysteine residues is currently unknown. Previous studies have connected acetyl-CoA synthetase with increased TAG production following nitrogen limitation due to increased acetyl-CoA accumulation and upregulation of *ACS2*, the gene encoding chloroplastic acetyl-CoA synthetase [85,86,87]. It is therefore possible that the reduction of cysteines is used to enhance the flux of acetyl-CoA production as the chloroplast remodels toward increasing neutral lipid production. Further *in vitro* investigation is necessary to determine the function of reversible thiol signaling in acetyl-CoA synthetase function and activity.

Chloroplastic ω-3-fatty acid desaturase (Cre01.g038600.t1.2) and chloroplastic ω-6-fatty acid desaturase (Cre13.g590500.t1.1) each showed significantly decreased cysteine oxidation, the former showing a FC_l2_ of −1.6 on C75 and C230 and the latter showing a FC_l2_ of −1.4 on C387. Our previous work associated an increase of reversible oxidation on C387 with the inhibition of target of rapamycin (TOR), a master-regulatory kinase whose inhibition causes phenotypic changes similar to those seen under nitrogen deprivation [33]. Similarly, our previous study revealed a 2.2 FC_l2_ on C653 of the phospholipase B-like protein (Cre03.g182750.t.2), whereas this current study showed a FC_l2_ of 3.3 on the same cysteine. While discrepancies related to the direction and/or magnitude of oxidative changes would be expected given fundamental differences in the two experiments, overlapping modulation on specific cysteine residues suggest functional importance that should be further pursued, especially as neither enzyme has been previously shown to be redox regulated.

## 3. Materials and Methods 

### 3.1. Chemicals and Reagents

All chemicals were purchased from Sigma-Aldrich (St. Louis, MO, USA) unless stated otherwise.

### 3.2. Cell Growth

Wild-type *Chlamydomonas reinhardtii* strain CC-2895 6145c mt- was purchased from the *Chlamydomonas* Resource Center (St. Paul, MN, USA). Four biological replicates for each condition were cultivated in foil-covered 250 mL flasks and grown photoheterotrophically in 100 mL of Tris-acetate-phosphate medium [88]. Cultures were maintained at 22 °C on an Innova 2000 platform shaker (New Brunswick Scientific, Enfield, CT, USA) at 120 rpm under constant 100 µmol m^−2^ s^−1^ illumination. Cells were grown to the mid-log phase (OD_750_ 0.4–0.5) before centrifuging for 5 min at 3220× *g*, discarding the supernatant, and washing with acetate-free media [17]. Following centrifugation, cells were resuspended in 100 mL of nitrogen deplete (made without NH_4_Cl) or replete media in 250 mL flasks and incubated in the growth chamber for 2 h. Cells were then harvested by centrifuging for 5 min at 3220× *g*, discarding the supernatant, and flash-frozen using liquid nitrogen. Cell pellets were stored at −80 °C until use. The experiment was repeated in its entirety (cell culturing through data analysis) four separate times. To minimize variance, data reported is from one of the experiments, during which all cells were grown and harvested on the same day.

### 3.3. Protein Extraction

Frozen cell pellets (0.6 g FW from 100 mL culture) were lysed in 10 mL phosphate-buffered saline (PBS) at pH 6.8 with 0.5% SDS, 0.1% Triton-X-100, and complete EDTA-free protease inhibitor cocktail (Roche, Basal, Switzerland). Reduced cysteines were blocked using 100 mM *N-*ethylmaleimide (NEM) dissolved in 50% ethanol. Samples were incubated end-over-end for 2 h at room temperature, covered from light to prevent artifactual oxidation and decrease NEM degradation due to light sensitivity [29]. Samples were then centrifuged at 3220× *g* to remove cellular debris before incubating the supernatant in 40 mL cold acetone to precipitate proteins for 1 h at −20 °C. Proteins were separated following a 5 min centrifugation at 3220× *g* and 4 °C before resuspending in 10 mL PBS pH 6.8 with 0.25% SDS and 4 M urea. Protein concentrations were quantified using the CB-X protein assay (G-Biosciences, St. Louis, MO, USA) according to the manufacturer’s protocols and samples were diluted to 1 mg⋅mL^−1^ with the resuspension buffer. Aliquots were taken for global proteomic (100 µg) and oxidized cysteine enrichment analysis (1 mg).

### 3.4. Global Proteomics

Aliquoted samples (100 µg) were incubated in constant darkness on a ThermoMixer (Eppendorf, Hamberg, Germany) at 25 °C and 1000 rpm. Samples were reduced with 10 mM dithiothreitol (DTT) for 30 min before adding 30 mM NEM for 30 min to alkylate cysteine residues. Samples were mixed with 1 mL of cold acetone to precipitate proteins and remove the DTT and NEM before being centrifuged for 5 min at 15,000× *g* and 4 °C. Pellets were resuspended in 500 µL of 50 mM Tris, pH 8 with 2 M urea and digested with 2.5 µg of Trypsin Gold (Promega, Madison, WI, USA) overnight (>16 h). The digestion was quenched using 20 µL of 5% trifluoroacetic acid (TFA) and desalted with solid-phase extraction (SPE).

### 3.5. Oxidized Cysteine Enrichment

Reversible thiol oxidation was enriched using an oxidized cysteine resin-assisted capture OxRAC) strategy, as described previously (Figure 1) [21,33]. Briefly, 1 mg of protein lysates were incubated with 10 mM DTT on a Thermomixer in constant darkness at 25 °C and 1000 rpm for 1 h in order to reduce all reversibly oxidized cysteines. DTT was then removed by precipitating proteins in 10 mL of cold acetone by incubating for 30 min at −20 °C. Following centrifugation (5 min, 3220× *g*, 4 °C), samples were resuspended in 1 mL of 50 mM Tris, pH 8 with 0.5% SDS and 4 M urea.

Thiopropyl Sepharose 6B (TPS6B; GE Healthcare, Pittsburgh, PA, USA) resin was rehydrated in water and washed with 50 mM Tris, pH 8 before suspending to a 100 mg/mL slurry. Each 1 mg *Chlamydomonas* proteome sample was added to 50 mg of TPS6B resin before incubating end-over-end for 2 h in the dark to covalently enrich proteins with reduced cysteines. Samples were then transferred to a MobiSpin column (Boca Scientific, Westwood, MA, USA) and nonspecifically bound proteins were removed by washing the resin (400 µL each) in 50 mM Tris, pH 8 with 0.5% SDS, 50 mM Tris, pH 8 with 2 M NaCl, and 80% acetonitrile with 0.1% TFA. The column was then equilibrated with 50 mM Tris, pH 8 before performing on-resin digestion of bound proteins using 250 µL of 50 mM Tris, pH 8 with 2.5 µg of Trypsin Gold. The digestion was incubated overnight (>16 h) on a covered Thermomixer at 25 °C and 1000 rpm. Following digestion, unbound peptides were separated from the cysteine-bound peptides by briefly centrifuging the spin columns for 15 s and discarded. Bound peptides were then washed (400 µL) using 50 mM Tris, pH 8 with 0.5% SDS, 50 mM Tris, pH 8 with 2 M NaCl, and 80% acetonitrile with 0.1% TFA. Bound cysteine-containing peptides were then eluted from the resin using 250 µL of 50 mM DTT in 50 mM Tris, pH 8 for 15 min in a Thermomixer at 25 °C and 1000 rpm. Samples were then centrifuged for 5 min at 1000× *g* before drying by vacuum centrifugation and desalting via SPE.

### 3.6. Solid-Phase Extraction

Samples were desalted using 50 mg Sep-Pak C18 cartridges (Waters) held in an SPE 24-position vacuum manifold (Phenomenex, Torrance, CA, USA) with a maximum flow rate of 1 drop/s. The resin was pre-eluted using 1 mL of 80% acetonitrile with 0.1% TFA before equilibrating with 1 mL of 0.1% TFA. Samples were acidified to pH 3 using 5% TFA and loaded onto the cartridges in two passes before washing with 1 mL of 0.1% TFA. Peptides were eluted using 1 mL of 80% acetonitrile with 0.1% TFA and dried by vacuum centrifugation.

### 3.7. LC–MS/MS Analysis

Samples were analyzed using an Acquity M-class UPLC system (Waters, Milford, MA, USA) coupled to a Q Exactive HF-X Hybrid Quadrupole-Orbitrap mass spectrometer (Thermo Scientific, Waltham, MA, USA). Mobile phase A consisted of water with 0.1% formic acid and mobile phase B was acetonitrile with 0.1% formic acid. Injections (4 µL) were made to a Symmetry C_18_ trap column (100 Å, 5 µm, 180 µm × 20 mm; Waters) with a flow rate of 5 µL/min for 3 min using 99% A and 1% B. Peptides were then separated on a HSS T3 C_18_ column (100 Å, 1.8 µm, 75 µm × 250 mm; Waters) using a linear gradient to 35% mobile phase B over 90 min at a flow rate of 300 nL/min.

The mass spectrometer was operated using a Nanospray Flex source in positive polarity mode with a spray voltage floating at 2.1 kV, a capillary temperature of 320 °C, and funnel RF level at 40. Data was acquired using a top 20 data-dependent acquisition mode with an isolation window of 1.5 *m*/*z*. The survey scans were collected with a scan range of 350–2000 *m*/*z* at a resolving power of 120,000 and an AGC target of 1 × 10^6^ with a maximum injection time of 50 ms. Fragmentation of precursor ions was performed by higher-energy collisional dissociation (HCD). MS/MS scans were performed with a scan range of 200–2000 *m*/*z* at a resolving power of 30,000 and an AGC target of 3 × 10^5^ with a maximum injection time of 100 ms. Precursors were subject to a dynamic exclusion of 10 s to provide increased coverage over a broader range of peptides.

### 3.8. Database Searching and Label-Free Quantification

Acquired spectral files (.raw) were imported into Progenesis QI for proteomics (Waters, version 2.0, Milford, MA, USA). Global and redox-enriched samples were analyzed separately. Peak picking sensitivity was set to a maximum of 5 and a reference spectrum was automatically assigned. Total ion chromatograms were aligned to minimize run-to-run differences in peak retention time. Each sample received a unique factor to normalize all peak abundance values resulting from systematic experimental variation. Alignment was validated (>80% score) and a combined peak list (.mgf) was exported for peptide sequence determination and protein inference by Mascot (Matrix Science, version 2.5.1, Boston, MA, USA). Database searching was performed against the Joint Genome Institute’s v.5.6 database (accessed Jan. 2020; 19,523 entries) appended with the NCBI chloroplast and mitochondrial databases (chloroplastic-NCBI: BK000554; mitochondrial-NCBI: NC_001638.1; 77 entries) and sequences for common laboratory contaminants (116 entries) [89,90]. Searches of MS/MS data used a trypsin protease specificity with the possibility of two missed cleavages, peptide/fragment mass tolerances of 15 ppm/0.1 Da, and variable modifications of acetylation at the protein N-terminus and oxidation at methionine. Cysteine alkylation with NEM (C_6_H_7_NO_2_, +125.0477) was included as a fixed modification for global analysis and a variable modification for oxidized cysteine enrichment experiments. Significant peptide identifications above the identity or homology threshold were adjusted to less than 1% peptide false discovery rate using the embedded Percolator algorithm and uploaded to Progenesis for peak matching [91]. Identifications with a score less than 13 were removed from consideration in Progenesis. For global proteomics, relative quantification was performed using the Hi-N setting with up to 3 peptides and protein grouping employed. Results were then exported from the ‘Review Proteins’ stage with both ‘Peptide Measurements’ and ‘Protein Measurements’ for reversible oxidation and global proteomics, respectively.

Data were parsed using custom scripts written in R programming for pre-processing and statistical analysis, as previously described [33]. To test for significantly changing oxidation and global abundance between the nitrogen deprived and replete conditions, a two-sided, equal variance *t*-test was performed. Only peptides with *p* < 0.05 after Benjamini–Hochberg correction and at least a two-fold change in oxidation were considered significant.

### 3.9. Data Availability 

The mass spectrometry proteomics data have been deposited to the ProteomeXchange Consortium via the PRIDE partner repository (PXD019491) [92].

## 4. Conclusions

While nitrogen deprivation in *C. reinhardtii* has been previously characterized to show large-scale physiological, proteomic, transcriptomic, and photosynthetic changes at the systems level, the mechanisms through which cultures rapidly respond and acclimate to starvation conditions is poorly understood. By quantifying changes in reversible oxidation 2 h following nitrogen deprivation, a framework for cell-wide oxidative regulation was observed. Significant oxidation increases occurred across the enzymes responsible for nitrogen cycling and reallocation, suggesting that short-term oxidation may upregulate nitrogen scavenging pathways while decreasing the flux into chloroplast. Additionally, significantly changing oxidation sites across the ferredoxin network as well as the chlorophyll biosynthesis pathway suggests that while photosynthetic flux and decreased chlorophyll accumulation does not occur until after 12+ h of nitrogen starvation, reversible oxidation may allow for rapid downregulation of pathway flux until autophagy takes over and unneeded protein pathways are dismantled. Further studies will benefit from molecular characterization of novel regulatory targets along photosynthetic and lipid metabolism pathways of *C. reinhardtii*.

## Figures and Tables

**Table 1:** *C. reinhardtii* was subjected to nitrogen deplete or replete conditions before harvesting and quantifying changes in reversible oxidation via LC–MS/MS. Significantly different peptides related to nitrogen allocation are presented. Each row represents a unique tryptic peptide containing an oxidized cysteine residue. Peptides originating from the same protein are grouped together by accession. Rows with more than one cysteine separated by commas denote multiple oxidized cysteines localized on the same peptide.

Accession	UniProt ID	Gene	Protein	Cys Site(s)	log_2_FC
Cre02.g113200.t1.1	A0A2K3E350	GLN1	Glutamine synthetase	C20	5.2
Cre09.g410750.t1.2	Q9ZR67	NII1	Nitrite Reductase	C509, C513	4.3
Cre09.g410850.t1.2	A0A2K3DFM1	NRT2.1	Nitrate transporter	C36	6.0
Cre10.g442800.t1.1	A8II65	XUV6	Xanthine/uracil permease	C13	5.5
				C47	8.5
Cre12.g504950.t1.2	A0A2K3D311	UOX1	Urate oxidase	C196	1.3
Cre12.g545101.t1.1	A0A2K3D6K5	XDH1	Xanthine dehydrogenase	C895	4.3
Cre12.g551352.t1.1	A0A2K3D647	LAO1	L-amino acid oxidase	C140	6.4
Cre12.g551353.t1.1	A0A2K3D651	LAO1	L-amino acid oxidase	C61	6.1
Cre13.g569850.t1.2	A8HSA2	AMT4	Ammonium transporter	C406	6.8
				C413	4.3
Cre16.g691750.t1.2	A8JBG4	AMI1	Formamidase	C74	7.8
				C363	3.4

**Table 2:** *C. reinhardtii* was subjected to nitrogen deplete or replete conditions before harvesting and quantifying changes in reversible oxidation via LC–MS/MS. Significantly different peptides related to photosynthesis are presented. Each row represents a unique tryptic peptide containing an oxidized cysteine residue. Peptides originating from the same protein are grouped together by accession. Rows with more than one cysteine separated by commas denote multiple oxidized cysteines localized on the same peptide.

Accession	UniProt ID	Gene	Protein	Cys Site(s)	log_2_FC
Cre01.g052250.t1.2	Q84XR9	TRXx	Thioredoxin x	C24	−4.3
Cre02.g082550.t1.2	Q84U73	ZEP1	Zeaxanthin epoxidase	C250	−1.3
Cre03.g183850.t1.2	Q2HZ21	FDX6	Ferredoxin	C228	−1.4
Cre03.g199800.t1.1	Q9FYU1	HYDA1	Fe-hydrogenase	C88	−1.6
				C225	−2.2
				C238	−2.6
Cre06.g272650.t1.2	Q75VY7	LHCA8	Chlorophyll a-b binding protein	C24	−1.3
Cre06.g306350.t1.2	Q2HZ24	FDX3	Ferredoxin	C138	−1.0
Cre07.g334800.t1.2	Q2HZ23	FDX4	Ferredoxin	C67, C72, C75	−1.2
Cre14.g626700.t1.2	A8IV40	PetF	Ferredoxin	C30	−1.6
				C115	1.2
Cre16.g658400.t1.2	A0A2K3CTD8	FDX2	Ferredoxin	C65	4.5

**Table 3:** *C. reinhardtii* was subjected to nitrogen deplete or replete conditions before harvesting and quantifying changes in reversible oxidation via LC–MS/MS. The significantly different peptides related to pigment biosynthesis are presented. Each row represents a unique tryptic peptide containing an oxidized cysteine residue. Peptides originating from the same protein are grouped together by accession. Rows with more than one cysteine separated by commas denote multiple oxidized cysteines localized on the same peptide.

Accession	UniProt ID	Gene	Protein	Cys Site(s)	log_2_FC
Cre01.g015350.t1.1	Q39617	POR1	Light-dependent protochlorophyllide reductase	C56	−4.5
Cre01.g015500.t1.2	A8HPJ5	CGL91	Predicted signaling protein	C10	1.3
Cre01.g043350.t1.2	Q9ZWM5	CAO1	Chlorophyll a oxygenase	C387	−2.3
Cre01.g050950.t1.2	A8HNE8	Unknown	Geranylgeranyl reductase	C115, C118, C125	−1.3
Cre02.g142266.t1.1	A5YU14	Unknown	CYP97A5	C231	−2.9
Cre07.g325500.t1.1	A8I7P5	CHLH1	Magnesium chelatase subunit H	C183	−1.8
				C598	−1.7
				C774	−1.8
Cre07.g342150.t1.2	Q9FPR7	HEMA1	Glutamyl-tRNA reductase	C129	−1.7
				C210	−1.8
				C267	−1.2
Cre07.g346050.t1.2	Q9LD46	CRD1	Magnesium-protoporphyrin IX monomethyl ester cyclase	C138	−2.7
Cre11.g467700.t1.1	A8JC21	UROD1	Uroporphyrinogen III decarboxylase	C299	−1.0
Cre12.g488350.t1.1	A0A2K3D2A6	Unknown	Uncharacterized protein	C578	−1.1
Cre16.g652000.t1.1	A0A2K3CSX1	Unknown	Carotenoid isomerase	C42	−1.5
Cre16.g663900.t1.2	A8JFB1	PBGD1	Porphobilinogen deaminase	C271	−1.7
NP_958412.2	P29683	chlN	Light-independent protochlorophyllide reductase subunit N	C250	−1.2
				C269, C273	−1.3
				C273	−1.2
				C291	−1.0
				C379	−1.4
NP_958360.1	P36437	chlB	Light-independent protochlorophyllide reductase subunit B	C169	−1.0
NP_958366.1	Q00469	chlL	Light-independent protochlorophyllide reductase subunit L	C36	−1.3
				C195	−1.9

**Table 4:** *C. reinhardtii* was subjected to nitrogen deplete or replete conditions before harvesting and quantifying changes in reversible oxidation via LC–MS/MS. The significantly different peptides related to lipid metabolism are presented. Each row represents a unique tryptic peptide containing an oxidized cysteine residue. Rows are grouped by protein identification. Rows with more than one cysteine denote multiple oxidized cysteines localized on the same peptide.

Accession	UniProt ID	Gene	Protein	Cys Site(s)	log_2_FC
Cre01.g038600.t1.2	A1E5M5	FAD7	Chloroplastic glycerolipid omega-3-fatty acid desaturase	C75	−1.6
				C230	−1.6
Cre01.g055408.t1.1	A8JCQ8	ACS2	Acetyl-CoA synthetase	C164	−1.0
				C594	−1.1
Cre02.g105200.t1.2	A8I478	Unknown	Saposin B domain-containing protein	C103, C109	1.6
Cre03.g182750.t1.2	A0A2K3DXV3	Unknown	Phospholipase B-like protein	C653	3.3
Cre03.g210513.t1.1	A8JBW0	Unknown	12-oxophytodienoate reductase 2	C23	−4.0
Cre05.g235700.t1.2	A8J8E3	Unknown	Saposin related protein	C213, C224	2.9
Cre13.g585301.t1.1	H1AFJ8	CPLD55	Monogalactosyldiacylglycerol synthase	C272	−1.0
Cre13.g590500.t1.1	O48663	FAD6	Omega-6-fatty acid desaturase	C387	−1.4

## Supplementary Materials

The following are available online at https://www.mdpi.com/2223-7747/9/6/784/s1, Table S1: Quantification of global protein samples following nitrogen deprivation, Table S2: Quantification of redox enriched peptides following nitrogen deprivation, Table S3: Oxidized proteins previously identified to be targets of thioredoxin, *S-*nitrosylation, and/or *S-*glutathionylation, Table S4: Significantly changing oxidized peptides relating to photosynthesis and/or chloroplastic regulation.
